# Clinicopathologic implications of Myb and Beta-catenin expression in adenoid cystic carcinoma

**DOI:** 10.1186/s40463-020-00446-1

**Published:** 2020-07-10

**Authors:** Susan Park, Manali Vora, Annemieke van Zante, Joseph Humtsoe, Hyun-Su Kim, Sue Yom, Shweta Agarwal, Patrick Ha

**Affiliations:** 1 Columbia University College of Dental Medicine, New York, NY USA; 2 Department of Otolaryngology Head and Neck Surgery, University of California San Francisco, San Francisco, California USA; 3 Department of Pathology, University of California San Francisco, San Francisco, California USA; 4 Department of Radiation Oncology, University of California San Francisco, San Francisco, California USA; 5 Department of Pathology, University of New Mexico School of Medicine, Albuquerque, NM USA

**Keywords:** Adenoid cystic carcinoma, Myb, Beta-catenin, Prognostic indicator

## Abstract

**Background:**

Adenoid cystic carcinoma (ACC) is the second most common malignancy of the salivary glands, accounting for ~ 1% of malignant tumors of the head and neck region and 10% of salivary gland neoplasms. Predicting the long-term outcomes of patients with ACC is still challenging, as reliable prognostic biomarkers are not available. Among salivary gland tumors, Myb overexpression is highly specific for ACC. In addition, the *MYB-NF1B* fusion translocation is a hallmark of ACC, and although the detection of this translocation does not appear to impact prognosis, the *MYB-NF1B* fusion is also implicated in MYB upregulation. Myb has recently been identified as an activator of the Wnt/β-catenin signaling pathway, and aberrant cytoplasmic expression of β-catenin has been observed in many salivary gland malignancies. In this study, we aim to analyze the impact of Myb and β-catenin expression on prognosis in ACC.

**Methods:**

A tissue microarray constructed from archival tissue from 64 patients with ACC was stained for Myb and β-catenin; both localization and intensity were evaluated. In parallel, we abstracted demographic data, tumor characteristics, survival data, and outcomes, including local recurrence, regional recurrence, and distant metastasis from the medical record. Statistical analysis was performed.

**Results:**

Our analysis supports that ACC patients negative for Myb by immunohistochemical methods have a higher risk of developing metastasis than patients with Myb staining (HR: 4.06, 95% CI: 1.02–14.96, *p*-value: 0.03). Although not statistically significant, cytoplasmic localization of β-catenin is may suggest a diminished rate of relapse-free survival (HR 2.45, 95%CI: 0.9–6.7, *p* = 0.08). Furthermore, Myb expression correlated with β-catenin expression, increasing 1.69 in staining intensity units with each increase in β-catenin staining intensity (*p*-value: 0.04).

**Conclusions:**

Our study suggests that Myb expression is protective; Myb positive patients have diminished risk of distant metastasis. In contrast, there is a trend towards increased hazard of death in ACC patients with cytoplasmic β-catenin expression. Additional analyses will be necessary to establish Myb and β-catenin as independent protective and adverse biomarkers, respectively.

## Background

Adenoid cystic carcinoma (ACC) is the second most common malignancy of salivary glands, accounting for approximately 1% of all malignant tumors of the head and neck region and 10% of all salivary gland neoplasms [1, 2]. A biphasic salivary gland neoplasm, ACC is composed of cells with myoepithelial and ductal phenotypes forming tubular, cribriform, and/or solid architectural patterns [3, 4]. Tumors are characterized by slow progression and frequent distant metastasis. Although ACC does not often spread to regional lymph nodes, approximately 52% of ACC patients develop distant metastasis with or without local-regional recurrence [5, 6].

The recommended treatment for ACC involves complete resection followed by postoperative radiotherapy for localized tumors; nonresectable cases are usually fatal [4, 7]. ACC is relatively refractory to conventional cytotoxic chemotherapy, perhaps by virtue of its slow growth kinetics [8, 9]. Predicting the long term outcome of individuals with ACC is challenging, as reliable prognostic biomarkers are not available. A better understanding of the pathophysiology and biomarkers in ACC is essential in predicting clinical outcomes.

Among salivary gland tumors, high nuclear Myb staining is specific for adenoid cystic carcinoma.

Myb is a transcription factor involved in cellular differentiation and proliferation and plays a role in oncogenesis in many types of cancer. The *MYB* locus (chr. 6) can undergo translocation with the *NF1B* locus (chr.9), and this *MYB-NF1B* t (6;9)(q22–23;p23–24) translocation is a molecular hallmark of ACC, found in over 50% of cases [10, 11].

Despite multiple studies, it remains unclear whether the *MYB-NFIB* fusion is associated with prognosis [12, 13]. On the other hand, overexpression of Myb has been found in 60% of fusion negative ACC, indicating that Myb overexpression may be a driver of pathogenesis in ACC independent of fusion status [12]. In colorectal cancers, c-Myb, a member of the MYB family, is a predictor of poor clinical outcome. Patients with low c-Myb demonstrated significantly decreased disease-free and overall survival [14]. Given these results, we sought to determine the prognostic significance of Myb expression in ACC.

β-catenin, a protein involved in cell-cell adhesion and gene transcription, has been proposed to have a role in tumor differentiation, invasion, and metastasis. When localized in the nucleus, β-catenin is involved in signaling through the canonical Wnt pathway and mediating gene expression. While at the plasma membrane, β-catenin and E-cadherin form a complex, maintaining cell-cell adhesion and mediating signal transduction important for cell proliferation and differentiation. In the setting of metastases, cytoplasmic localization of β-catenin is thought to be involved in the epithelial-mesenchymal transition, based on studies of breast and salivary gland cancers [15–17].

In head and neck squamous cell carcinoma (HNSCC), it has been suggested that β-catenin mainly functions in adhesion rather than cell signaling [18]. Recent studies have demonstrated reduced membrane localization and increased cytoplasmic localization of β-catenin in malignant salivary gland tumors when compared to benign salivary gland tumors. Cytoplasmic localization of β-catenin may be indicative of a lack of differentiation or invasive potential in the malignant tumors [19]. Cytoplasmic localization of β-catenin in non-small cell lung cancer (NSCLC) also correlates with poor prognosis [20].

Further supporting a role for β-catenin in malignancies, aberrant expression of β-catenin has been found to promote the pathogenesis of gastric cancer and breast cancer [21, 22]. Recent studies have demonstrated that abnormal dephosphorylation of β-catenin, increased nuclear translocation, and reduced proteasome-mediated degradation leads to the increased levels β-catenin in ACC [23].

To date, the clinical significance of the overexpression of Myb and the role of β-catenin localization in ACC is unknown. Here, we sought to evaluate the prognostic significance of β-catenin and Myb expression in a cohort of patients with ACC. Our results suggest that a high level of Myb is associated with diminished risk of metastasis, while cytoplasmic localization of β-catenin is may suggest decreased survival.

## Methods

### Patient selection

Data was collected and analyzed in accordance with established guidelines and were reviewed and approved by the University of California-San Francisco Institutional Review Board. Seventy-three patients were identified through a search of the UCSF pathology database for ACC of the head and neck between 1991 and 2015. Eleven patients were excluded from the study: Myb and β-catenin staining were not interpretable for two patients, and nine patients were excluded from the study due to the lack of clinical follow-up data. After histologic confirmation of the diagnosis by a board-certified pathologist (AvZ), sixty-two patients with a non-zero follow-up data had intact, adequate tissue samples for analysis. A retrospective chart review was completed to determine the clinical variables for this cohort.

### Data collection

#### Tissue microarray (TMA) construction

From the 64 patients, representative duplicate or triplicate 2 mm punches of formalin-fixed paraffin-embedded tumors were assembled into a tissue microarray (TMA). The TMA blocks were subsequently sectioned and stained for Myb and β-catenin.

#### Beta-catenin immunohistochemistry

Immunohistochemical staining for β-catenin was performed on the Leica BOND III Automated stainer. Briefly, 4 μm TMA sections were deparaffinized with xylene and ethanol. Antigen retrieval was performed using citrate buffer (pH 6.0) for 20 min. Sections were incubated with clone 14/β-catenin (BD Transduction Lab, Franklin Lakes, NJ) diluted 1:20 for 15 min. External positive and negative controls were performed.

β-catenin staining was scored based on the intensity of cytoplasmic staining using a 3-point scale from 0 to 3: (0: absent; 1: weak; 2: moderate; 3: intense). Scoring was performed by a board-certified pathologist (AvZ) blinded to patient outcomes. The mean score of the multiple tumor samples was utilized for statistical analysis.

#### Myb immunohistochemistry

Immunohistochemical staining for Myb was performed using the Dako EnVision+ System (Dako, Glostrup, Denmark). Briefly, 4um TMA sections were deparaffinized with xylene and ethanol. Heat-induced epitope retrieval was completed with EnVision Module at 97 °C for 20 min in pH 9.0 buffer. Endogenous peroxidase activity was blocked with Dako EnVision Flex. Tissue sections were then incubated with an anti-Myb monoclonal antibody (Genetex, Irvine, CA). The reaction was visualized using the Dako EnVision Flex+ System. External positive and negative controls were performed.

Myb was scored based on both % cells staining and the intensity of staining. The % of cells showing any staining was visually assessed along with the intensity of Myb staining using a 3-point scale: (0: absent; 1: weak; 2: moderate; 3: intense). Scoring was performed by two independent board-certified pathologists (AvZ and SA) with discrepant scores re-examined by both observers to achieve a consensus score.

#### Clinical data

Demographic data, tumor characteristics, as well as outcomes, including local recurrence, regional recurrence, and distant metastasis, were obtained from the UCSF electronic medical record. Time to local recurrence and distant metastasis were defined as the time between such events and the primary treatment. Overall survival (duration of total follow up) was defined as the time from diagnosis to death by any cause.

#### Statistical analysis

All statistical analyses were carried out with STATA 14. Event time was determined based on the date of diagnosis or surgery to the time of an event (metastases, recurrence, or death). Prognostic value of Myb expression, β-catenin expression, and other clinicopathological characteristics were evaluated using the Cox proportional hazard model. Hazard ratios are presented with their 95% confidence intervals (CIs). Additionally, the regression model was fit to assess the association between Myb expression and β-catenin expression (measured through respective staining intensities) in the tumor samples evaluated. Statistical tests were two-sided at the 5% level of significance.

## Results

### Patient and clinical characteristics

The mean age of the patients was 55.0 years. The subjects were comprised of 25 men and 39 women. The majority (62.5%) of the patients were white, with 26.6% Asian and 10.9% African American or other. Most patients (57.8%) were nonsmokers.

As expected, most patients (73.4%) received additional treatment after surgical resection (Table 1). Of the 64 patients who underwent primary surgery, 47 patients had postoperative adjuvant radiotherapy, concurrent chemoradiotherapy, or chemotherapy.

**Table 1:** Patient and tumor sample characteristics

Variable	Overall ***N*** = 64	***p***-value
Age at diagnosis (in years)	55 (+/−15.1)	0.3
Sex		0.6
Male	25 (39%)	
Female	39 (61%)	
Race ^a^		0.7
White	40 (62.5%)	
Asian/PI	17 (26.6%)	
African American and Other	07 (10.9%)	
Smoking history ^b^		0.1
No	37 (57.8%)	
Yes	20 (31.3%)	
Missing	07 (11.0%)	
Tumor stage ^c^		0.005
Early (Stage 2 or earlier)	22 (31.3%)	
Late (Stage 3 or later)	36 (56.3%)	
Missing	08 (12.5%)	
Tumor location		0.04
Major salivary gland	19 (29.7%)	
Minor salivary gland	45 (70.3%)	
Perineural invasion ^d^		0.1
Yes	41 (64.1%)	
No	11 (17.9%)	
Missing	12 (18.8%)	
Margin status ^e^		0.2
Negative	06 (09.4%)	
Positive	45 (70.3%)	
Missing	13 (20.3%)	
Dominant pattern		0.3
Tubular/Cribriform	43 (67.2%)	
Solid	15 (20.3%)	
Missing	08 (12.5%)	
Tumor necrosis	13 (20.3%)	0.3
Treatment ^f^		0.3
Surgery	17 (26.6%)	
Surgery +RT/C or RT/C alone	47 (73.4%)	
Missing	01 (1.34%)	

^a^There were very few African American patients in our sample; thus, we combined them with people who disclosed their race as other, who were also few.

^b^Patients were categorized as smokers if they reported current or formerly smoking.

^c^Tumor stages were determined clinically using TNM tumor classification.

^d^Perineural invasion was determined histologically.

^e^All patients in our study received some treatment. We dichotomized them in two categories: those patients who received surgical treatment and those who underwent radiation therapy (RT) and chemotherapy (C) in addition to surgery or independent of surgical treatment.

The majority (56.3%) of patients in the study were diagnosed with cancers at stage 3/4, and 70% had a tumor originating in the minor salivary glands. Approximately 67.2% of cases exhibited predominantly tubular growth patterns, while 20.3% demonstrated predominantly cribriform or solid growth patterns on histology. Microscopic examination of the primary tumor tissue revealed that perineural invasion was found in the majority of cases (64.1%).

### Prognostic factors affecting overall survival

β-catenin staining were not interpretable for two patients in our sample. They were excluded from statistical analysis evaluating association between β-catenin expression and survival/ metastasis/ recurrence in ACC patients. Similarly, an additional patient was excluded due to uninterpretable MYB staining from model evaluating MYB expression as a prognostic factor. The mean clinical follow-up time (diagnosis to death) was 58 months (0–303 months). Median follow up was 41.8 months. Follow up data was right skewed, i.e., more people had a shorter follow up. In the univariate analysis (Table 2), age at diagnosis (HR: 1.03, 95% CI:1.0–1.1, *p*-value: 0.03), smoking history (HR: 2.9, 95% CI:1.1–8.2, *p*-value: 0.03) and clinical stage at diagnosis (HR: 2.21, 95% CI:1.2–4.2, *p*-value: 0.02) were the only significant predictors of survival.

**Table 2:** Univariate Analysis: Outcomes of patients with ACC based on prognostic factors

Overall survival ^a^	N	HR*	95% CI	***p***-value
Myb Staining (high vs. low)	63	1.05	0.45–2.44	0.9
Myb staining (present vs. absent)	63	1.57	0.63–3.8	0.4
Myb % tumor average (continuous)	64	0.99	0.98–1.01	0.6
Myb % tumor average (high vs. low)	64	1.05	0.45–2.44	0.9
ß-catenin cytoplasmic staining	62	2.45	0.9–6.7	0.08
Age at diagnosis	64	1.03	1.0–1.1	0.03
Race	63	0.92	0.5–1.9	0.8
Sex	64	1.06	0.4–2.6	0.8
Smoking history	57	2.9	1.1–8.2	0.03
Stage at diagnosis	56	2.21	1.2–4.2	0.02
Margin status	51	1.62	0.21–12.5	0.6
Dominant pattern	56	1.64	0.6–4.4	0.3
Treatment	64	1.31	0.6–3.1	0.5
**Metastasis**^**b**^		HR*	**95% CI**	***p*-value**
Myb Staining (high vs. low)	58	2.40	0.62–9.36	0.17
Myb staining (present vs. absent)	58	4.06	1.02–14.96	0.03
Myb % tumor average (continuous)	59	0.98	0.96–1.01	0.2
Myb % tumor average (high vs. low)	59	2.27	0.58–8.90	0.2
ß-cat cytoplasmic staining	57	1.35	0.39–4.67	0.64
Age at diagnosis	59	1.01	0.97–1.06	0.52
Race	58	0.36	0.08–1.57	0.18
Sex	59	0.46	0.09–2.22	0.34
Smoking history	54	0.72	0.15–3.59	0.69
Stage at diagnosis	51	1.62	0.79–3.32	0.19
Margin status	46	–	–	–
Dominant pattern	51	–	–	–
Treatment	59	1.22	0.34–4.37	0.76
**Recurrence**^c^		HR*	**95% CI**	***p*-value**
Myb Staining (high vs. low) ^a^	58	1.61	0.61–4.25	0.33
Myb staining (present vs. absent) ^b^	58	1.05	0.36–3.05	0.91
Myb % tumor average (continuous)	59	1.01	0.19–1.02	0.23
Myb % tumor average (high vs. low)	59	0.50	0.18–1.36	0.16
ß-cat cytoplasmic staining ^c^	57	1.88	0.64–5.54	0.25
Age at diagnosis	59	1.03	0.99–1.07	0.13
Race	58	1.22	0.55–2.74	0.62
Sex	59	0.43	0.14–1.35	0.15
Smoking history	54	1.99	0.67–5.95	0.22
Stage at diagnosis	51	3.31	1.02–10.69	0.04
Margin status	46	–	–	–
Dominant pattern	51	1.56	0.32,7.54	0.58
Treatment	59	1.08	0.33–3.54	0.89

The N in the table is presented as a separate column because each predictor had a differential amount of missing, and thus the sample size is not consistent among models fit to assess the different associations.

*: Hazard Ratios

** Follow-up information regarding overall survival, metastasis, and recurrence was missing for 9 patients, 11 patients, and 12 patients, respectively. Additionally, ß-cat staining information was unavailable for 2 patients, and Myb staining information was unavailable for 1 patient.

No estimates have been presented for margin status and dominant histologic pattern. All cases of recurrence and metastasis had a positive margin status. Given the small sample size and missing patient information for dominant histologic pattern, a survival model could not be fit to accurately analyze the data to provide conclusive results.

^a^The average follow-up time for patients in whom overall survival was evaluated was 58.3 months, ranging from 0 to 303.1 months

^b^The average follow-up time for patients in whom metastasis was evaluated was 51.5 months, ranging from 0 to 262.5 months

^c^The average follow-up time for patients in whom overall recurrence was evaluated was 50 months, ranging from 0.07–244.5 months

Regarding β-catenin, staining was predominantly cytoplasmic in all cases. The risk of dying increased by 2.5 fold in patients with higher intensity of staining when compared to patients with one unit lower intensity of β-catenin staining (HR 2.45, 95% CI: 0.9–6.7, *p*-value: 0.08), although the association did not reach statistical significance. We also evaluated the prognostic value of Myb by the percentage of cells staining, Myb by staining intensity, sex, race of the patient, the dominant histopathological pattern of the tumor, margin status, and treatment received. Although not statistically significant, our findings suggest that race and sex of the patient, margin status, the dominant histological pattern of tumor, and treatment may be potential predictors of survival (Table 2).

### Prognostic factors impacting metastasis

Information on metastasis and time to metastasis was available for 58 patients. The mean follow-up time was 51.5 months (0–262.5 months), median follow up was 41.4 months. Myb staining was a significant factor affecting the risk of distant metastasis. ACC patients with no Myb staining had a higher risk of developing metastasis than patients with any Myb staining (HR: 4.06, 95% CI: 1.02–14.96, *p*-value: 0.03). All patients with a positive tumor margin experienceed metastasis at follow up.

### Prognostic factors impacting recurrence

Information on recurrence (local, regional, and distant) and time to recurrence were collected from 57 patients. The mean time to recurrence was 50 (0.07–244.5) months whereas median time to recurrence was 38.1 months. Only the stage at diagnosis was significantly predictive of recurrence (HR: 3.31, 95% CI:1.02–10.69, *p*-value: 0.04). All patients with a positive tumor margin had recurrence at follow up.

### Association between Myb and β-catenin expression

Linear regression was performed to evaluate the association of Myb expression with β-catenin staining intensity. A significant regression equation was found (F (1,67) = 4.60, *p*-value: 0.04) with an R^2^ of 0.06. Myb expression increased 1.69 in staining intensity units for each unit of β-catenin staining intensity.

## Discussion

Our cohort includes 64 ACC patients with predominantly of minor salivary gland tumors (69.6%). As would be expected, these tumors frequently demonstrated perineural invasion (78.9%). The stage at diagnosis was a strong independent predictor of recurrence and poor overall survival, as has been found in previous studies [13]. Age at diagnosis and smoking history were also significant predictors of overall survival in this cohort. All cases of recurrence and metastasis had a positive margin status. Evaluation of Myb and β-catenin expression by immunohistochemistry support that any Myb expression is associated with diminished risk of metastasis (HR: 4.06, 95% CI: 1.02–14.96, *p*-value: 0.03) suggesting that Myb expression is a valuable as a prognostic biomarker. However, the effect size reported is not precise. True effect in the population could be either very small (1.02) or very large (~ 15). Future studies with larger cohorts need to confirm our hypothesis and generate a more precise effect estimate of association between MYB and ACC metastasis.

Higher intensity of cytoplasmic β-catenin staining was suggestive of a higher risk of death. β-catenin staining intensity was assessed visually using a 3 point scale. Although not statistically significant, we observed that each unit increase in staining intensity of β-catenin was associated with a 2.5-fold increased risk of dying (HR 2.45, 95% CI: 0.9–6.7, *p-*value: 0.08). Furthermore, a linear regression analysis demonstrated a significant association between Myb and β-catenin expression. Each staining intensity unit increase in β-catenin resulted in an increase in 1.69 units in the average Myb staining intensity. The mechanism behind the interaction between Myb and β-catenin in ACC are unclear; however, c-Myb, a member of the MYB family, has been found to be an activator of the Wnt/β-catenin signaling pathway, promoting invasion and metastasis of breast cancer [24]. Myb and β-catenin have not been previously evaluated as biomarkers with prognostic implications in ACC.

### MYB

*MYB-NF1B* t (6;9)(q22–23;p23–24) translocation results in disruption of the *MYB* 3′ UTR, which contains a negative regulatory site required for *MYB* downregulation. The loss of negative regulation, coupled with the juxtaposition of super-enhancer regions of *NFIB* and *MYB* results in the overexpression of Myb [11].

Previous Myb expression studies demonstrated that 89% of tumors, regardless of fusion status, overexpressed Myb RNA [25], and approximately 61–82% of analyzed ACC tumors stained positive for Myb [12, 25, 26]. In agreement with previous findings, the prevalence of Myb staining in this study was 69%. The overexpression of *MYB* is often associated with the t (6;9) translocation producing the *MYB-NFIB* fusion protein, which is thought to be the hallmark of ACC. However, previous studies show *MYB-NFIB* is not a strong predictor of prognosis [12, 13]. In addition, approximately 70% of fusion negative ACC overexpress Myb, suggesting a role in pathogenesis.

Given the rarity of ACC and resultant limits on sample size, we could not analyze Myb expression as a factor independent of other variables including age at diagnosis, stage at diagnosis, and smoking history. Nevertheless, our study supports that Myb expression is protective against the development of metastatic ACC. A higher-powered analysis is necessary to establish Myb as an independent biomarker.

### β-Catenin

Expression and localization of β-catenin are thought to have a role in tumor differentiation, metastasis, and invasion. Studies have found metastasis is promoted through the dephosphorylation of β-catenin by PP2A, which inhibits β-catenin degradation and increases β-catenin expression in ACC [23].

Similar to previous findings [27], immunostaining for β-catenin localized to the cytoplasm in all of our tumor samples. The significance of the distribution is not entirely clear; however, other studies have observed cytoplasmic localization of β-catenin in malignant salivary gland tumors [24]. Our findings suggest that cytoplasmic localization of β-catenin may be an indicator of aggressive behavior and metastatic potential in malignant salivary gland tumors.

ACC is a rare cancer. One of the biggest limitations of this study is the small sample size. As a result, the analyses may be under-powered to detect the true associations in the population. Future studies with a larger cohort will be necessary to establish Myb and β-catenin as independent prognostic biomarkers.

## Conclusion

The absence of reliable prognostic markers presents a challenge in predicting long-term outcomes in patients with ACC; however, we believe our study provides insight on potential prognostic factors for the assessment of clinical outcome. We report in this study on the association of Myb and β-catenin expression with different clinical outcome in adenoid cystic carcinoma. Overexpression of Myb and β-catenin has been demonstrated previously in the setting of ACC, but the significance has thus far been unclear. Our study found that expression of Myb is associated with a decrease in risk of distant metastasis, whereas increased cytoplasmic expression of β-catenin might be associated with higher mortality among ACC patients. Moreover, an association between Myb and β-catenin expression is evident. We hypothesize that both Myb and β-catenin are important prognostic markers in ACC patients.

## Data Availability

All data generated or analyzed during this study are included in this published article.

## Abbreviations

TMA: Tissue microarray ACC Adenoid cystic carcinoma β-catenin Beta-catenin
